# Development and validation of Work-Related Activities during Non-Work Time Scale (WANTS) for doctors

**DOI:** 10.1371/journal.pone.0241577

**Published:** 2020-11-18

**Authors:** Mohd Fadhli Mohd Fauzi, Hanizah Mohd Yusoff, Nur Adibah Mat Saruan, Rosnawati Muhamad Robat

**Affiliations:** 1 Department of Community Health, Faculty of Medicine, Universiti Kebangsaan Malaysia, Cheras, Kuala Lumpur, Malaysia; 2 Ministry of Health Malaysia, Federal Government Administrative Centre, Putrajaya, Malaysia; 3 Occupational and Environmental Health Unit, Selangor State Health Department, Shah Alam, Selangor, Malaysia; Nord University, NORWAY

## Abstract

Work-related activities during non-work time may influence the intershift recovery of post-work fatigue. Currently there is no valid and reliable scale available to measure the frequency for such activities among doctors. Therefore, this study aims to develop and validate ‘Work-Related Activities during Non-Work Time Scale’ (WANTS) that measure the frequency of work-related activities during non-work time for doctors. This was a scale development and validation study among doctors involving item generation, content and construct validation, and reliability assessment. 23-item seven-point Likert-type scale was developed through deductive (literature search) and inductive (interview with source population, authors’ experiences, and expert opinion) methods. The content-validated scale was pre-tested, and the improved scale was subsequently administered to randomly-selected 460 doctors working at public hospital setting. Response rate was 77.76% (n = 382). Initial exploratory factor analysis (EFA) with principal axis factoring (PAF) using varimax rotation revealed unstable six-factor structure consisting of 17 variables; thus, we tested one- to six-factor model, and found that four-factor model is the most stable. Further analysis with principal component analysis (PCA) with a single component on each factor found that 17-variables four-factor model is stable. These factors were labelled as ‘work-related thought’, ‘work-to-home conversation’, ‘task spillover’ and ‘superior-subordinate communication’. It showed good internal consistency with overall alpha value of 0.837. The scale is thus valid and reliable for measuring the frequency of each construct of work-related activities during non-work time among doctors.

## Introduction

Work-related activity during non-work time is a type of demand that consume personal energy resources and consequently influences fatigue recovery [1]. Fatigue and recovery are closely related concepts [2, 3]. On one hand, fatigue refers to the state of reduced capacity, either physically or psychologically, to perform work-related activity as a result of depleted personal energy resources [2, 3]. On the other hand, recovery refers to the unwinding of fatigue through energy restoration to its pre-demand level in the absence of demand such as work-related activity [2–6]. Central to the concept of recovery is the idea that employees need breaks from the work demand to recover from effort-driven work-related fatigue in order to function optimally on the next working day [7, 8]. Based on the effort-recovery model and conservation of resources theory, inadequate recovery will cause fatigue accumulation and subsequently commence a cycle of accumulated fatigue which eventually lead to irreversible fatigue [3, 9, 10].

Intershift activity, or activity during non-work period, influences recovery [11]. Generally, intershift activities were classified into work-related activity and other non-work activities such as physical exercise, social activity, household chores, social interaction with family, hobby or creative activity. Sonnentag conceptualized work-related activity during non-work time as high duty, that is, activity that consumes personal energy resources and impedes recovery [11]. Demerouti *et al*. listed work-related activity as one of the intershift activities that potentially inhibits recovery [12]. Matthews *et al*. extend the discussion on work-related activities during non-work time by conceptualizing it as interdomain transition, that is, home-to-work or home-to-family transition [13]. It refers to the frequency of behavioral action of role shifting made from home domain to work domain during non-work period [13]. For example, during non-work period, individual should assumes family role at home domain; however, due to some circumstances, he/she has shifts his/her role to employee role at work domain during non-work time. Higher frequency of work-related activities exerts more efforts [9] and drain more energy resources which consequently hinder recovery [14]. Apart from that, multiple studies specifically focus on the use of communication technology such as smartphones for work-related purposes during non-work time [15–19]. In those studies, the measures include the usage, duration, and frequency of work-related smartphone use during non-work time [15–19]. As far as we concern, there is limited study on work-related activities during non-work time specifically among doctors population despite multiple available studies on fatigue and its consequences among this fatigue-prone population.

Doctors are among the main workforce in health service delivery; and their nature of work exposed them to various fatigue-prone factors at work such as demanding job and long work hours [20]. It is thus not surprising that the prevalence of fatigue among doctors is worrying. For instance, in Saudi Arabia, Arab & Khayyat found that 54% of 102 Anesthesia residents were at risk of fatigue [21]. In the United Kingdom (UK), 84.2% of 2,231 Anesthesia trainees were in fatigue state during post-night shift [22]. Recently, a national survey of consultant anesthetists and pediatric intensivists in the UK and Ireland found that 91% of them reported having work-related fatigue [23]. Additionally, in China, Tang *et al*. found that 78.8% of 1,729 full-time doctors at 24 tertiary hospitals had high level of occupational fatigue [24]. A study among intensive care unit doctors providing 24-hour resident cover on a 12-hour day and 12-hour night shift roster found that they experience high level of fatigue in both day and night shift; however, the night shift lead to worsening fatigue [25].

High prevalence of fatigue among doctors is a major occupational issue that adversely affect the doctors, patients, and organization. Fatigue doctor is at high risk of commuting accident [26] and work-related accident such as needlestick injury [27]. Fatigue doctors may jeopardize patients’ care such as decision fatigue [28] and serious diagnostic errors [29]. Other fatigue consequences include medication errors, adverse health and wellbeing, work-life dissatisfaction, low quality of life, job dissatisfaction, poor cognitive and poor skill performance [30]. The inadequate unwinding of post-work fatigue, or lack of fatigue recovery, during non-work period cause the doctors to work on the subsequent shift with fatigue residue [31] and put them at risk of fatigue consequences [30]. Moreover, if the recovery process during non-work time is persistently inadequate, fatigue may accumulate, and consequently progresses into non-reversible chronic fatigue [32].

It is therefore crucial to ensure adequate recovery during non-work period as it is significantly related with fatigue. Recovery can be classified into process and outcome [6, 33]. Recovery process refers to the activities (such as work-related activities, household activities, social activities) and experiences (such as psychological detachment from work, relaxation, mastery) [6, 11, 33] that may lead to a change in functioning and strain level. Recovery as an outcome implies an employee’s psychophysiological state that is reached after a recovery period such as state of recovery, work engagement, cortisol level, and proactive behavior [6, 33]. We will focus on recovery as a process, and specifically on the activities during non-work period. In general, studies shown that work-related activities hamper recovery process [6, 33].

The spillover of the work-related demand into home domain during non-work time potentially disrupts recovery experiences of psychological detachment from work [11]. A two-wave panel study among 230 healthcare workers at three general hospitals in Netherland found that those who were involved in work-related activities during off-job hours reported being less able to psychologically detach from work [32]. Recent study found that work-related smartphones use during non-work time was negatively associated with psychological detachment from work [15]. This was also supported by previous researches which found significant negative relationship between the usage, duration and frequency of work-related smartphone use during non-work time with psychological detachment from work [16–19]. However, limited study was found to specify the type and context of work-related smartphone use, whether it is an unanticipated command from superior, or unwelcoming informal discussion with colleague. Work-related ruminative thoughts, such as thoughts on the incident of being violated at work, mistakes being done at work, patient-related conditions, and task-related aspects may as well hamper the recovery process during non-work time [20, 34–36]. Adverse events at work such as violence is highly prevalent among healthcare workers [37–39] and is associated with intrusive thoughts [40]. Other work-related activities include pressures related to unfinished or forthcoming task [20].

Measurement for work-related activities during non-work time is mostly unspecific. Most of the available instruments measure time spent for work-related activity or frequency of involvement in work-related activity during non-work time by cumulating it as one single indicator. For instance, Sonnentag measures work-related activity by asking the participants to report in diary the time they start and end the activity such as finishing or preparing for work duties and use the cumulative time spent as an indicator [11]. Similarly, Garrick *et al*. measures work-related activities during non-work time by defining it as hours spent on school-related tasks outside of paid working hours such as lesson preparation and assignment marking [41]. In contrast, Matthews *et al*. develop and tested a sub-scale of home-to-work transition among heterogenous group of workers (i.e. management, business, financial operations, administrative, etc.) that measure the frequency of work-related activities during non-work time using 6-item 7-point Likert scale such as receiving calls from co-workers while at home or changing plans with family to meet work-related responsibilities activities [13]. Nevertheless, the existing instruments did not examine the dimensions of each work-related activities which may exert different effect against recovery process. In addition, despite extensive research on the work-related activities during non-work time among employees, little is known about work-related activities being done by healthcare workers, particularly doctors.

As doctors’ duty involved high-intensity job demand and fatigue-prone schedule with potential adverse fatigue consequences, it is crucial to examine their work-related activities during non-work time. Therefore, this study aims to develop and validate the questionnaires that measures the frequency of work-related activities during non-work time among doctors in Malaysia.

## Materials and methods

### Study design

This was a study on development and validation of scale related to work-related activities during non-work time among doctors. This study consisted of four phases: (a) item generations, (b) content validation, (c) construct validation, and (d) reliability assessment.

### Study setting

This study was conducted in year 2019 at seven core clinical disciplines (i.e. internal medicine, surgery, orthopedic, pediatric, obstetrics and gynecology, anesthesiology and psychiatry) from seven public hospitals under Ministry of Health Malaysia. This study was a continuation study to identify latent constructs of work-related activities during non-work time following cross sectional findings on fatigue and recovery among Malaysian doctors [42].

### Participants / sampling

Our reference population is medical doctors working at public hospital in Malaysia. Our source population was medical doctors working at seven public hospitals in the state of Selangor. We included all Malaysian medical officers (i.e. medical doctors who has completed housemanship training but not yet specialist) who have been working at current workplace for at least one month. We excluded post-graduate medical officers and those who have been diagnosed as sleep disorder of psychiatric illness. The study involved six occupational health experts with at least five years experiences in occupational health, and 14 medical doctors to represent reference population during item-generation and content-validation phases [43, 44]. For construct validation phase, we distributed the questionnaire to 460 randomly-sampled eligible study population based on the number of items i.e. 23 items (participant-to-item ratio of 20:1) [44].

### Study phase

#### Item generation

We developed the initial instrument using a combination of deductive and inductive methods [44]. Deductive method involves scoping literature search specifically on type of activities during non-work time being done by general employees [43, 44]. On the other hand, inductive methods include interview with source population, authors’ experiences, and expert opinion [43, 44]. The items generated from both methods were finally combined into one list.

For deductive method, literature search was conducted from Web of Science Core Collection with aims to identify publications that measure any work-related activities during non-work time among employees. The search strategy was: ("fatigue" OR "recovery") AND ("activity" OR "activities") AND ("work" OR "works") AND ("intershift" OR "non-work") across timespan from 1998 to 2018. To further expand the findings, we also conducted literature search from the listed references for each relevant paper.

To enrich the items generated, we purposively approached fourteen medical doctors from one public hospital in Selangor representing target population [44]. They consist of one senior and one junior medical doctors from each core clinical disciplines who differ in their gender. They were asked to exhaustively describe any work-related activities that they do during their non-work time. All authors, who are also medical doctors, also added their own experiences on work-related activities during non-work time throughout their career as medical doctors. Next, three occupational health experts were purposively approach to list and describe any work-related activities during non-work time that they do or know of [44]. All of them are qualified occupational health doctors registered with Department of Safety and Health (DOSH) Malaysia and had at least five years of experience in occupational health services. One of them works in private sector, while the others work in government sector.

#### Content validation

We assessed the initial instrument for content to ensure adequate coverage of work-related activities during non-work time and clarity to ensure comprehensibility through discussion among authors. Items with similar meaning were grouped together to avoid redundancy. The wordings for each item and instructions for the scale were designed to be simple, clear, specific, and non-ambiguous [45]. Subsequently, another three occupational health experts were approached and asked on relevance and clarity of each item [46] which is within recommended number of experts for content validation [47]. They are also qualified occupational health doctors registered with DOSH Malaysia and had at least five years of experience in occupational health services. Similarly, one of them works in private sector, while the others work in government sector. Content validity was evaluated quantitatively by them using content validity index (CVI) and content validity ratio (CVR) [48].

CVI quantify the content validity based on expert ratings of relevance and clarity. It can be computed at item level (I-CVI) or scale level (S-CVI). For I-CVI relevancy, experts were asked to rate the relevance of each item on a 4-point scale: “1 = not relevant”, “2 = somewhat relevant”, “3 = quite relevant”, and “4 = very relevant” [49, 50]. For each item, the I-CVI is computed as the number of experts giving a rating of either 3 or 4, divided by the number of experts, that is the proportion in agreement about relevance. The cut-off points for accepted I-CVI is 1.00 [51], and if the value is below 1.00, the item is eliminated [50]. For the S-CVI, we calculated the average I-CVI across items, which refers as S-CVI/Ave [52]. S-CVI/Ave was calculated by taking the sum of the I-CVIs divided by the total number of items. The acceptable value was set at 0.90 [53]. Similar approach was done for I-CVI clarity using the following 4-point Likert scale: “1 = not clear”, 2 = item need some revision”, “3 = clear but need minor revision”, and “4 = very clear”.

CVR measures the essentiality of an item [54, 55]. For CVR, experts were asked to specify whether an item is necessary to represent work-related activities during non-work time among doctors by using 3-point Likert scale: “1 = not necessary”, “2 = useful but not essential”, and “3 = essential”. The acceptable value of content validity ratio is set at 1.00 [50]. The formula of content validity ratio is CVR = (N_e_—N/2)/(N/2), in which the N_e_ is the number of experts indicating "essential" and N is the total number of experts [50].

The improved instrument consists of 23 items with seven-point Likert scale i.e. 0 (never), 1 (less than once per month), 2 (once per month), 3 (more than once per month), 4 (once a week), 5 (more than once a week), and 6 (daily). This instrument was pre-tested through cognitive interview [56] among same fourteen medical doctors as in item-generation phase. The aims of cognitive interview were to evaluate how participants comprehend, interpret and answer the questions [56]. They were asked to provide constructive feedback on comprehensibility of the instructions and each item in the scale such as formats, wordings, arrangement, and others [56]. They were also asked whether they understood the item the way the researcher intended [56]. Based on their feedback, the instrument was improved to ensure face validity.

#### Construct validation and reliability

Construct validity was assessed with the use of exploratory factor analysis (EFA), while reliability was measured by the use of internal consistency and item-total correlation [45]. Based on the rule of thumb [44, 45, 57], we distributed the instrument among randomly-selected 460 medical officers.

EFA was conducted using Principal Axis Factoring (PAF) with varimax rotation [58, 59]. We developed bivariate correlation matrix to identify multicollinearity and removed pair of items with bivariate correlation scores greater than 0.8, if any [60]. We also assessed the determinant of the matrix, which should be greater than 0.00001 [58]. We assessed the adequacy of the sample size using Kaiser-Meyer-Olkin measure of sampling adequacy (KMO) test which measuring the data quality for the factor analysis, with a minimum acceptable score of 0.5 [61]. Bartlett’s test of Sphericity was conducted to verify if there is a relationship between the variables. Item with communality less than 0.2 was removed one-by-one. We then assessed the cross loading, in which item that cross loaded on more than one factor were removed in turn, starting with the highest ratio of loadings item. Subsequently, item with factor loading less than 0.5 were removed in turn, starting with the lowest factor loading item [59]. The number of factors to be retained was initially decided by cuts off factor eigenvalues less than one [58, 59]. Retained factors were assessed to ensure that they have at least three items with a loading greater than 0.5 and did not cross load on other factors [58, 59].

Since the retained factors did not fulfil the requirement [59], we tested for various fixed-factor model by examining the correlation matrix determinant, KMO statistic, Bartlett’s test of Sphericity, communality, total variance explained, number of items with factor loading more than 0.4 and 0.5, and number of factors with less than three items with loading more than 0.4 and 0.5. All retained factors should have at least three items with a loading greater than 0.4 with at least 50% of total variance explained by the retained factors [62]. Once deciding on number of factors to be retained, we conducted PCA without rotation on each single factor to ensure each group unique items will load on each respective factor [58]. Subsequently, we re-develop bivariate correlation matrix to assess the intercorrelation. A reliability analysis was then undertaken for each scale [58, 59]. Finally, we made decision on final model and determine the label for each dimension.

### Ethical consideration

This research was registered under National Medical Research Register (NMRR) (NMRR-19-1249-48464). Ethical approval was obtained from Medical Research & Ethics Committee (KKM/NIHSEC/P19-1326). Consent for participation was obtained prior to interviews or survey participation. Their personal information was strictly confidential. The anonymity of participants was ensured by not collecting any identifiable data such as name, identity card number and phone number.

## Results

### Item generated

Literature search revealed 20 results; however, only five articles were relevant. From these five papers, we generate several items which include ‘work-related thoughts related to official task and adverse events at work’ [12, 63], ‘work-related use of communication technology such as internet, smartphone, and laptop’ [63], ‘doing unfinished task at home’ [41, 64], ‘preparing for next working day’ [41, 64], and ‘locum’ which can be attributed as work-related volunteer activity [65]. Further literature search through articles listed in these five papers revealed similar types of activities such as ‘finishing or preparing for work duties’ [11], ‘doing one’s private administration’ [11], ‘work-related thoughts related to official task’ [20] ‘work-related thoughts related to adverse events at work’ [34–36, 40] and ‘physical and virtual communications’ with various parties’ [16–19], and ‘locums’ [20].

As for the interview, we revealed items related to meeting, training, task, and discussion, either physically or virtually through digital communication medium. For instance, one participant stated that he sometime had to attend meeting or training during post-call period despite supposed to be at home. Some participants also mentioned that they have to finish their tasks at workplace beyond official working hour as they were expected to attend and settle managing new cases who arrived even five minutes before their official working hour finished. It is also not uncommon that several participants described that they involved in work-related discussion initiated through online medium such as WhatsApp after working hours. Almost all of them described that they had at least once involved in preparing work-related presentation or completing patient-related reports at home during non-work time.

To expand the items, we also add on other items based on our own experiences as doctors such as informal conversation about work with family members and handling of work-related digital media. For instance, one author had involved in handling Facebook related to work during non-work time to update the information or checking email inbox. Other authors commonly talk with their spouse or parents on their work on daily basis, either face-to-face or by phone. Expert opinions involving occupational health physician listed almost similar type of activities as above. However, they suggest that all relevant types of activities were expanded by categorizing it based on types (i.e. work-related thought, task, communication), medium (i.e. physical /face-to-face or virtual) and interaction group (i.e. self, superior, colleague, family).

### Content validation

Initial pool consisted of 30 items were content-validated by three occupational health experts. 23 items had I-CVI and CVR of 1.00. The I-CVI for another 7 items ranged between 0.00 and 0.67, while their CVR ranged between -1.00 to 0.33. The S-CVI for relevance of this 30-item pool were 0.844. Initial pool of 30 items was then reduced to 23 items which demonstrate I-CVI and CVR of 1.00 and resulting in S-CVI of 1.00. Following interview with fourteen medical doctors, re-wording for some items based on feedback were done to ensure comprehensibility. Table 1 presented initial scales containing 23 items which were classified based on the types of activity (work-related thoughts, task, communication), medium (psychological, physical, virtual) and interaction groups (superior, colleague, clients, family, self).

**Table 1:** Initial scale with 23 items.

Items	Types	Medium	Interaction Group
Work-related thoughts on mistakes being done at work either deliberately or unintentionally	Thought	Psychological	Self
Work-related thoughts on unfinished formal task	Thought	Psychological	Self
Work-related thoughts on incident of being scolded or violated at work	Thought	Psychological	Self
Work-related thoughts on upcoming formal task and assignments	Thought	Psychological	Self
Work-related thoughts on patients / clients	Thought	Psychological	Self
Informal, work-related discussions with colleagues at the workplace	Communication	Physical	Colleague
Face-to-face communications with patients / clients	Communication	Physical	Clients
Informal, work-related discussions with colleagues virtually	Communication	Virtual	Colleague
Performing official tasks at the workplace	Task	Physical	Mixed
Informal conversation about work with spouse or partner virtually	Communication	Virtual	Family
Informal face-to-face conversations about work with spouse of partner	Communication	Physical	Family
Virtual conversations about work with parents	Communication	Virtual	Family
Face-to-face conversations about work with parents	Communication	Physical	Family
Formal work assignments by employer via telephone call	Communication	Virtual	Mixed
Formal work assignments by employer via text or email	Communication	Virtual	Superior
Attending formal, work-related meeting physically at the workplace	Task	Physical	Superior
Attending formal, work-related meeting virtually	Task	Virtual	Superior
Doing official tasks at home	Task	Physical	Mixed
Handling work-related email / website / social media	Task	Virtual	Mixed
Attending work-related upskill training at workplace	Task	Physical	Mixed
Virtual communication with patients/clients	Communication	Virtual	Clients
Locum at private health facilities	Task	Physical	Mixed
Locum at government health facilities	Task	Physical	Mixed

### Construct validation

#### Descriptive analysis

The response rate was 83.04% (n = 382). The mean age of participants was 31.21 (SD = 3.488) years with average work tenure as doctors of 4.60 (SD = 3.075) years. The majority of them were female (n = 242, 63.4%) and married (n = 253, 66.2%). Most of the participants had no children (n = 206, 53.9%), followed by one children (n = 89, 23.3%), and two children (n = 62, 16.2%). All of them reported using WhatsApp as digital work-related communication medium. 38.2% (n = 146) also used e-mail as second medium for work-related digital communication.

Table 2 listed 23 work-related activities during non-work time, which were ranked based on the mean score. Thought-related activities during non-work time dominated the top sixth ranking, which indicates frequency of at least once a week.

**Table 2:** Work-related activities during non-work time.

Variables	Mean (SD)	Rank
Work-related thoughts on patients / clients	4.80 (1.696)	1
Performing official tasks at the workplace	4.62 (1.907)	2
Informal, work-related discussions with colleagues virtually	4.45 (1.788)	3
Face-to-face communications with patients / clients	4.32 (2.425)	4
Work-related thoughts on upcoming formal task and assignments	4.13 (1.993)	5
Work-related thoughts on unfinished formal task	4.12 (1.934)	6
Informal, work-related discussions with colleagues at the workplace	3.77 (2.102)	7
Work-related thoughts on mistakes being done at work either deliberately or unintentionally	3.63 (1.943)	8
Informal face-to-face conversations about work with spouse of partner	3.55 (2.352)	9
Work-related thoughts on incident of being scolded or violated at work	3.30 (2.014)	10
Informal conversation about work with spouse or partner virtually	3.04 (2.388)	11
Formal work assignments by employer via text or email	2.83 (1.965)	12
Doing official tasks at home	2.81 (1.858)	13
Formal work assignments by employer via telephone call	2.68 (1.779)	14
Face-to-face conversations about work with parents	2.64 (1.948)	15
Virtual conversations about work with parents	2.01 (2.082)	16
Handling work-related email / website / social media	1.84 (1.919)	17
Attending formal, work-related meeting physically at the workplace	1.66 (1.493)	18
Attending work-related upskill training at workplace	1.30 (1.471)	19
Virtual communication with patients/clients	0.99 (1.624)	20
Attending formal, work-related meeting virtually	0.81 (1.480)	21
Locum at private health facilities	0.80 (1.476)	22
Locum at government health facilities	0.46 (1.059)	23

#### Exploratory factor analysis

Bivariate correlation matrix showed none of the pairs had bivariate correlation scores greater than 0.8; thus, no item was removed. An EFA was then conducted on this initial 23-item scale by using a PAF technique with a Varimax rotation. First observation showed that KMO test was 0.803 with significant Bartlett’s Test, and one item with communality less than 0.2 (i.e. ‘locum at private health facilities’) was removed. Re-observation showed that KMO test was 0.806 with significant Bartlett’s Test, and one item with communality less than 0.2 (i.e. ‘locum at government health facilities’) was removed. Next re-observation showed that KMO test was 0.809 with significant Bartlett’s Test, and one item with communality less than 0.2 (i.e. ‘attending work-related upskill training at workplace’) was removed. Another re-observation showed that KMO was 0.808 with significant Bartlett’s Test, and no item had communality less than 0.2. This led to a solution comprising of six factors, in which three factors have loadings of 0.4 on at least 3 items, while another three factors have only two items with loadings of 0.4, and several items in the rotated factor matrix cross loaded on more than one factor. These cross loaded items were removed in turn, starting with the item with the highest ratio of loadings on the most variables with the lowest highest loading. First item being removed was ‘doing official task at home’ which loaded on three factors with factor loading of 0.334, 0.383 and 0.354. Re-observation led to six factors solution. Items with highest factor components much less than 0.5 were then removed in turn starting with lowest factor loading i.e. ‘virtual communication with patients/clients’ (factor loading: 0.365). Re-observation was done, and another factor component with factor less than 0.5 was removed i.e. ‘handling work-related email / website / social media’ (factor loading: 0.470). This eventually yielded a solution with 17 variables with six factors (Table 3). Two factors have at least three items with a loading greater than 0.5, but another four factors only have two items.

**Table 3:** The improved six-factor model.

Items	Factor					
	1	2	3	4	5	6
Work-related thoughts on upcoming formal task and assignments	.770					
Work-related thoughts on mistakes being done at work either deliberately or unintentionally	.767					
Work-related thoughts on unfinished formal task	.743					
Work-related thoughts on incident of being scolded or violated at work	.718					
Work-related thoughts on patients / clients	.577					
Informal, work-related discussions with colleagues at the workplace		.773				
Face-to-face communications with patients / clients		.638				
Performing official tasks at the workplace		.582				
Informal, work-related discussions with colleagues virtually		.564				
Informal face-to-face conversations about work with spouse of partner			.875			
Informal conversation about work with spouse or partner virtually			.849			
Formal work assignments by employer via text or email				.759		
Formal work assignments by employer via telephone call				.739		
Face-to-face conversations about work with parents					.754	
Virtual conversations about work with parents					.705	
Attending formal, work-related meeting virtually						.748
Attending formal, work-related meeting physically at the workplace						.630

Note: Extraction method: Principal Axis Factoring; Rotation Method: Varimax with Kaiser Normalization; Extraction criterion: Eigenvalues higher than one. Total variance explained by extracted components: 73.908%; KMO = 0.779; Bartlett’s test: χ^2^ = 2820.333, p < 0.001

Table 4 showed the result of further testing for different fixed-factor model using forced-factor extraction method, and four-factor model was identified as the most stable.

**Table 4:** n-factor model.

Parameter	n-Factor Model					
	1	2	3	4	5	6
Rotation	None	Varimax	Varimax	Varimax	Varimax	Varimax
Technique	Principal axis factoring					
Correlation matrix determinant	0.001					
KMO Statistic	0.779					
Bartlett’s Test of Sphericity	Significant					
Communality <0.2	None					
Total Variance Explained, %	29.263	42.891	53.996	61.670	67.922	73.908
Variables loading factors >0.4	11	13	14	15	14	13
Variables loading factors >0.5	8	10	11	12	12	10
Factors with < 3 items with loading >0.4	0	0	0	0	2	4
Factors with < 3 items with loading >0.5	0	0	0	0	3	4
Stability^a^	No	No	No	Yes	No	No

^a^ Stability is determined based on correlation matrix determinant (<0.0001), KMO statistic (> 0.5), Bartlett’s test of sphericity (significant), communality <0.2 (none), total variance explained (>60.00%), variables loading factors >0.4 (most of the items have loading >0.4), and number of items with loading >0.4 per factors (each factor has >3 items with loading >0.4)

We decided to use the four-factor model. Subsequent PCA without rotation on each single factor was done (Table 5). PCA for each single factor fulfilled the following criteria: (a) correlation matrix determinant (<0.0001), (b) KMO statistic (> 0.5), (c) Bartlett’s test of sphericity (significant), (d) communality <0.2 (none), (e) total variance explained (>60.00%), (f) variables loading factors >0.4 (all items), and (g) number of items with loading >0.4 per factors (all factors have >3 items with loading >0.4).

**Table 5:** PCA analysis on each single factor of four-factor model with factor loadings.

Items	Factors			
	1	2	3	4
Work-related thoughts on mistakes being done at work either deliberately or unintentionally	.825			
Work-related thoughts on unfinished formal task	.829			
Work-related thoughts on incident of being scolded or violated at work	.777			
Work-related thoughts on upcoming formal task and assignments	.837			
Work-related thoughts on patients / clients	.737			
Informal conversation about work with spouse or partner virtually		.861		
Informal face-to-face conversations about work with spouse of partner		.766		
Virtual conversations about work with parents		.737		
Face-to-face conversations about work with parents		.680		
Informal, work-related discussions with colleagues at the workplace			.843	
Face-to-face communications with patients / clients			.782	
Informal, work-related discussions with colleagues virtually			.690	
Performing official tasks at the workplace			.761	
Formal work assignments by employer via telephone call				.822
Formal work assignments by employer via text or email				.761
Attending formal, work-related meeting physically at the workplace				.749
Attending formal, work-related meeting virtually				.610

A reliability analysis showed that all four factors were considered acceptable as each had at least three component scores greater than 0.7 (Table 6).

**Table 6:** Final four-factor model with reliability analysis.

Item	Factor				Inter-Item Correlation	Alpha	
	1^a^	2 ^b^	3 ^c^	4 ^d^		Factor	Total
Work-related thoughts on mistakes being done at work either deliberately or unintentionally	.784				0.715	0.861	0.837
Work-related thoughts on unfinished formal task	.739				0.713		
Work-related thoughts on incident of being scolded or violated at work	.736				0.649		
Work-related thoughts on upcoming formal task and assignments	.720				0.722		
Work-related thoughts on patients / clients	.579				0.598		
Informal conversation about work with spouse or partner virtually		.909			0.706	0.759	
Informal face-to-face conversations about work with spouse of partner		.722			0.559		
Virtual conversations about work with parents		.547			0.525		
Face-to-face conversations about work with parents		.473			0.463		
Informal, work-related discussions with colleagues at the workplace			.751		0.670	0.770	
Face-to-face communications with patients / clients			.593		0.586		
Informal, work-related discussions with colleagues virtually			.549		0.473		
Performing official tasks at the workplace			.530		0.571		
Formal work assignments by employer via telephone call				.655	0.627	0.720	
Formal work assignments by employer via text or email				.620	0.546		
Attending formal, work-related meeting physically at the workplace				.573	0.523		
Attending formal, work-related meeting virtually				.428	0.367		

Note:

^a^work-related thought;

^b^work-to-home conversation;

^c^task spillover;

^d^superior-subordinate communication

## Discussion

We established a valid and reliable 17-items four-factor scale specifically for doctors. The top 13 activities, based on ranking that was set according to mean, were statistically-selected by EFA into these 17-items scale, while bottom two items were statistically-removed. All these 17 items were statistically-grouped into four latent constructs, which are labelled as ‘**work-related thought**’, ‘**work-to-home conversation**’, ‘**task spillover**’, and ‘**superior-subordinate communication**’. By identifying latent construct of WANTS, researchers may be able to quantify frequency for each construct activities, determine their association with health outcome and planning the intervention.

The items were generated by combining inductive and deductive technique, and involving target population, expert judgment and authors’ own experiences. These ensure optimum coverage with appropriate relevance of context [44]. The use of EFA for exploratory construct validation which assumes that any indicator or variable may be associated with any factor is appropriate [44, 58]. The use of EFA to assess the underlying factor structure and refine the item pool is recommended as a precursor to CFA [44, 46, 58, 59]. As the name suggested, it is not based on any prior theory, but solely depends on the statistical test. Thus, it may or may not been fully explainable by current available theory. Based on EFA, we able to obtain four latent construct which is fully explainable.

**‘Work-related thought’** is broadly defined as repetitive and intrusive thinking in response to negative moods or life situations related to work [66]. As far as our knowledge, there is limited similar construct of work-related thought existed in the current prevailing literature. We acknowledged the presence of construct measuring work rumination as psychological activity [67–70], however, it does not cover all type of work-related activities aspects during non-work time such as work-related conversation or physical work task. Most of thought, or rumination, measurement instruments also mostly developed to measure its association with affect such as depression, anxiety, and phobia [67–70], but very limited on fatigue and recovery. This gap in the work-related thought literature limits the ability of researchers to understand the role that work-related thinking plays during intershift period in fatigue recovery particularly among doctors. Through the development of a valid and reliable measure of WANTS, the research in this area can be extended to provide greater clarity on the ways work-related thought is related to recovery.

**‘Work-to-home conversation’** is not uncommon, in which people often bring their work-related issues from one domain into the other [71]. It is a type of work-to-family spillover, which generally can occurs when behaviors, moods, stress, and emotions from work are transferred to the family domain [72]. Grzywacz & Marks indicate that there are four distinct work-family spillover experiences: negative and positive spillover from work to family and family to work [73]. There is limited study to indicate whether work-to-home conversation is a negative or positive work-related spillover, particularly among doctors. The role of content or context or conversation may play a role, however we did not specify them into this scale; instead, we just generalized it as work-to-home conversation. Thus, it may be necessary to reassess the indication to specify the content and context, however it need trade off with lengthy questionnaires.

**‘Task spillover’** refers to work-related task spillover from work hour’s domain into off-job hour’s domain, either at workplace or at home, either physically present or through virtual means, and either involve directly or indirectly person at work such as colleague and clients or patients [74–76]. For example, ‘doing work task at workplace’ during off-job hours is not uncommon. The scheduling system with lack of overlapping hours for shift handover push the doctors to work extra hours unpaid to ensure proper shift handover and safety of the patients. In addition, doctors are expected to welcome all the patients or clients until end of shift without any buffer hour to settle the pending task. For example, supposed the doctors finish their job on-schedule at 1700H, but the clients came at 1650H; the doctors have to attend to this patient comprehensively from history taking, physical examination, lab or imaging investigation, and others, due to lack of overlapping hours for shift handover. Similarly, after handover the patients including the newly-came patients, their colleague may call or text them to clarify the cases or update the cases. Consequently, the doctors are doing extra unpaid job either physically or virtually, which consume their internal resources, and put them at high risk of fatigue [10, 77]. Additionally, their off-job hours are being cut indirectly, and put them at risk of lack of recovery [10, 77].

**‘Superior-subordinate communication’** refers to the interactions between organizational leaders such as medical specialist or administrative people with their subordinates, either virtually or physically present at workplace, to achieve personal and organizational goals [78]. Communication can be solely transmission, solely reception, or exchange of messages between superior and subordinate [79]. This downward communication type is used to coordinate efforts and activities, to instruct, to direct, or to explain company decisions [79]. Subordinates react most effectively to those matters that they judge to be of greatest personal interest to the boss [80]. Thus, it is not uncommon that subordinate responds to their superior during off-job hours and predispose them to work-related activities.

This newly-developed instrument has several similarity and differences with previously-developed instruments. This instrument quantifies the work-related activities during non-work time based on the frequency, which is similar with multiple instruments in previous studies [11, 13, 41]. In addition, this instrument covers both activities related to psychological and behavioral aspect of recovery process during non-work time [13]. This coverage is also consistent with the concept of interdomain transition that highlighted the transition of physical and cognitive from home domain to work domain during non-work time [13]. Despite some similarities, the advantage of this instrument is on its differences with previously-developed instruments. First, this instrument combines three aspects of work-related activities namely types of activity (i.e. work-related thoughts [12, 34–36, 63], task [11, 12, 20, 41, 64], communication [15–19, 63]), medium (i.e. psychological [12, 34–36, 63], physical [11, 12, 20, 41, 64], virtual [15–19, 63]) and interaction groups (i.e. superior, colleague, clients, family, self) before statistically determine and validate its latent construct. Second, this instrument distinguished four latent constructs of work-related activities during non-work time i.e. work-related thoughts, work-to-home conversation, task spillover, and superior-subordinate communication. Third, this instrument is developed specifically for medical doctors whom their intershift recovery is rarely explored despite high risk of having fatigue from multiple work exposure. Nevertheless, this instrument still needs further refinement and validation among target population in different workplace and geographical setting.

There are several limitations in our study. First, this is the first and only exploratory validation conducted on this newly-developed instrument; thus, it is not yet ready to be used to test a theory. Qualitative studies involving focus group discussion, in-depth interview or diary documentation among target population should be considered to ensure exhaustive list of work-related activities during non-work time is established. Second, study location involved hospitals in urban setting, which may significantly differ from rural or suburban setting which may influence type of work-related activities during non-work time. This scale should be tested on similar target population, but different work and home environment, including suburban and rural setting. Third, the conduct of item generation among medical doctors only from one public hospital may limit the number of unique items generated as participants from same centre may experience similar work-related activities during non-work time. However, effort to ensure heterogeneity was done by purposively selecting different gender and seniority from each clinical discipline from that one hospital.

## Conclusions

The development of WANTS enabled the measurement of work-related activities during non-work time among doctors using a single close-ended Likert scale that includes four identifiable domains, namely work-related thoughts, work-to-home conversation, task spillover, and superior-subordinate communication. WANTS is found to be content- and constructively valid and reliable. This custom-designed scale for measuring work-related activities during non-work time is beneficial for research purposes and organizational practices. On one hand, researcher could quantify work-related activities during non-work time based on specific latent construct instead of using a single-item general work-related intershift activity involvement or listing each item independently which cause redundancy. On the other hand, organization could determine which aspect of work factors that spillover into the home domain and manifested as work-related activities during non-work time, and further planning the organizational intervention on work-home interface. However, further assessment is still needed to refine the construct among medical doctors in different setting.

## Supporting information

S1 Questionnaire Berikut ialah kenyataan tentang aktiviti-aktiviti berkaitan kerja yang anda lakukan ketika bukan waktu bekerja. Bulatkan pada satu nombor antara 0 hingga 6 yang paling menggambarkan kekerapan anda melakukan aktiviti-aktiviti berkaitan kerja tersebut ketika bukan waktu bekerja. *(The following are statements regarding work-related activities that you do beyond official work hours*. *Please circle on the number between 0 (never) to 6 (daily) that represents the frequency you do work-related activities beyond official work hours)*.
(DOCX)

## References

[1] Bakker AB, Demerouti E. The Job Demands-Resources model: State of the art. Journal of Managerial Psychology. 2007;22(3): 309–328. 10.1108/02683940710733115

[2] Phillips RO. A review of definitions of fatigue—and a step towards a whole definition. Transportation Research Part F: Traffic Psychology and Behaviour. 2015;29: 48–56. 10.1016/j.trf.2015.01.003

[3] Meijman TF. Mentale Belasting en werkstress: Een arbeidspsychologische benadering [Mental workload and job stress: A work psychological approach]. 1989 Assen/Maastricht, The Netherlands: Van Gorcum

[4] Geurts SA, Sonnentag S. Recovery as an explanatory mechanism in the relation between acute stress reactions and chronic health impairment. Scand J Work Environ Health. 2006;32(6):482–92. 10.5271/sjweh.1053 17173204

[5] Sonnentag S, Natter E. Flight attendants’ daily recovery from work: Is there no place like home? Int J of Stress Manag. 2004;11(4):366–391. 10.1037/1072-5245.11.4.366

[6] Sonnentag S, Venz L, Casper A. Advances in recovery research: What have we learned? What should be done next? J Occup Health Psychol. 2017;22(3): 365–380. 10.1037/ocp0000079 28358572

[7] Steed LB, Swider BW, Keem S, Liu JT. Leaving work at work: a meta-analysis on employee recovery from work. Journal of Management. 2019 10.1177/0149206318786781 30774168 PMC6343426

[8] ten Brummelhuis LL, Bakker AB. Staying engaged during the week: the effect of off-job activities on next day work engagement. J Occup Health Psychol. 2012;17(4):445–55. 10.1037/a0029213 22799771

[9] Meijman T.F. & Mulder G. 1998 Psychological aspects of workload In Drenth P/J.D., Thierry H. & de Wolff C.J. (eds.). Handbook of Work and Organizational Psychology. Handbook of Work and Organizational: Work Psychology. 2nd Ed pp. 5–33. UK: Psychology Press/Erlbaum Taylor & Francis 10.1037//1076-8998.3.2.109

[10] Hobfoll SE, Halbesleben J, Neveu J-P, Westman M. Conservation of resources in the organizational context: the reality of resources and their consequences. Annual Review of Organizational Psychology and Organizational Behavior. 2018;5(1):103–128. 10.1146/annurev-orgpsych-032117-104640

[11] Sonnentag S. Work, recovery activities, and individual well-being: a diary study. J Occup Health Psychol. 2001;6(3): 196–210. 10.1037/1076-8998.6.3.196 11482632

[12] Demerouti E., Bakker A. B., Geurts S. A. E., & Taris T. W. (2009). Daily recovery from work-related effort during non-work time In Sonnentag S., Perrewé P. L., & Ganster D. C. (Eds.), Research in occupational stress and well-being: Vol. 7. Current perspectives on job-stress recovery (p. 85–123). JAI Press/Emerald Group Publishing 10.1108/S1479-3555(2009)0000007006

[13] Matthews RA, Barnes-Farrell JL, Bulger CA. Advancing measurement of work and family domain boundary characteristics. J Vocat Behav. 2010;77(3): 447–460. 10.1016/j.jvb.2010.05.008

[14] ten Brummelhuis LL, Trougakos JP. The recovery potential of intrinsically versus extrinsically motivated off-job activities. J Occup Organ Psychol. 2014;87(1): 177–199. 10.1111/joop.12050

[15] Cambier R, Derks D, Vlerick P. Detachment from work: a diary study on telepressure, smartphone use and empathy. Psychol Belg. 2019;59(1):227–245. 10.5334/pb.477 31328018 PMC6625542

[16] van Laethem M, van Vianen AEM, Derks D. Daily fluctuations in smartphone use, psychological detachment, and work engagement: the role of workplace telepressure. Front Psychol. 2018;9:1808 10.3389/fpsyg.2018.01808 30319504 PMC6166396

[17] Mellner C. After-hours availability expectations, work-related smartphone use during leisure, and psychological detachment: the moderating role of boundary control. Int J of Workplace Health Manag. 2016;9(2): 146–164.

[18] Richardson K, Thompson C. High tech tethers and work-family conflict: a conservation of resources approach. Eng Manag Res. 2012;1(1): 29–43. 10.5539/emr.v1n1p29

[19] Derks D, van Mierlo H, Schmitz EB. A diary study on work-related smartphone use, psychological detachment and exhaustion: examining the role of the perceived segmentation norm. J Occup Health Psychol. 2014;19(1): 74–84. 10.1037/a0035076 24447222

[20] British Medical Association. Fatigue and sleep deprivation—the impact of different working patterns on doctors. 2018. https://www.bma.org.uk/-/media/files/pdfs/collective%20voice/policy%20research/education%20and%20training/fatigue-sleep-deprivation-briefing-jan2017.pdf?la=en

[21] Arab AA, Khayyat HY. Risk of fatigue among anesthesia residents in Saudi Arabia. Saudi Med J. 2017;38(3): 292–296. 10.15537/smj.2017.3.17511 28251225 PMC5387906

[22] McClelland L, Holland J, Lomas JP, Redfern N, Plunkett E. A national survey of the effects of fatigue on trainees in anaesthesia in the UK. Anaesthesia. 2017;72(9): 1069–1077. 10.1111/anae.13965 28681546

[23] McClelland L, Plunkett E, McCrossan R, Ferguson K, Fraser J, Gildersleve C, et al A national survey of out-of-hours working and fatigue in consultants in anaesthesia and paediatric intensive care in the UK and Ireland. Anaesthesia. 2019;74(12): 1509–1523. 10.1111/anae.14819 31478198

[24] Tang C, Liu C, Fang P, Xiang Y, Min R. Work-related accumulated fatigue among doctors in tertiary hospitals: a cross-sectional survey in six provinces of China. Int J Environ Res Public Health. 2019;16(17): 3049 10.3390/ijerph16173049 31443480 PMC6747540

[25] Bihari S, Venkatapathy A, Prakash S, Everest E, McEvoy RD, Bersten A. ICU shift related effects on sleep, fatigue and alertness levels. Occup Med (Lond). 2020;70(2): 107–112. 10.1093/occmed/kqaa013 31974569

[26] Barger LK, Cade BE, Ayas NT, Cronin JW, Rosner B, Speizer FE, et al Extended work shifts and the risk of motor vehicle crashes among interns. N Engl J Med. 2005;352(2): 125–34. 10.1056/NEJMoa041401 15647575

[27] Motaarefi H, Mahmoudi H, Mohammadi E, Hasanpour-Dehkordi A. Factors associated with needlestick injuries in health care occupations: a systematic review. J Clin Diagn Res. 2016;10(8):IE01–IE04. 10.7860/JCDR/2016/17973.8221 27656466 PMC5028444

[28] Persson E, Barrafrem K, Meunier A, Tinghög G. The effect of decision fatigue on surgeons’ clinical decision making. Health Econ. 2019;28(10): 1194–1203. 10.1002/hec.3933 31344303 PMC6851887

[29] Landrigan CP, Rothschild JM, Cronin JW, Kaushal R, Burdick E, Katz JT, et al Effect of reducing interns’ work hours on serious medical errors in intensive care units. N Engl J Med. 2004;351(18): 1838–48. 10.1056/NEJMoa041406 15509817

[30] Gates M, Wingert A, Featherstone R, Samuels C, Simon C, Dyson MP. Impact of fatigue and insufficient sleep on physician and patient outcomes: a systematic review. BMJ Open. 2018;8(9): e021967 10.1136/bmjopen-2018-021967 30244211 PMC6157562

[31] Meijman TF. Belasting en herstel: een begrippenkader voor arbeids-psychologisch onderzoek van werkbelasting [Effort and recuperation: a conceptual framework for psychological research of workload] In: Meijman TF, editor. Mentale belasting en werkstress: een arbeidspsychologische benadering [Mental workload and job stress: a work psychological approach]. Assen/Maastricht, Netherlands: Van Gorcum; 1989 pp. 5–20.

[32] de Jonge J, Shimazu A, Dollard M. Short-term and long-term effects of off-job activities on recovery and sleep: a two-wave panel study among health care employees. Int J Environ Res Public Health. 2018;15(9): 2044 10.3390/ijerph15092044 30231562 PMC6164214

[33] Sonnentag S, Geurts SAE. Methodological issues in recovery research In: Sonnentag S, Perrewé PL, Ganster DC, editors. Current perspectives on job-stress recovery: research in occupational stress and well-being. Oxford, UK: Emerald Publishing Group; 2009 pp. 1–36.

[34] Querstret D, Cropley M. Exploring the relationship between work-related rumination, sleep quality, and work-related fatigue. J Occup Health Psychol. 2012;17(3): 341–53. 10.1037/a0028552 22746369

[35] Niven K, Sprigg CA, Armitage CJ, Satchwell A. ruminative thinking exacerbates the negative effects of workplace violence. J Occup Organ Psychol. 2013;86: 67–84. 10.1111/j.2044-8325.2012.02066.x

[36] Lunardo R, Saintives C. Feeling a little guilt but ruminating a lot: how indulgence impacts guilt and its consequences In: Kubacki K, editor. Ideas in marketing: finding the new and polishing the old. Developments in Marketing Science: Proceedings of the Academy of Marketing Science. Cham, Switzerland: Springer; 2015 Pp. 805–813.

[37] Behnam M, Tillotson RD, Davis SM, Hobbs GR. Violence in the emergency department: a national survey of emergency medicine residents and attending physicians. J Emerg Med. 2011;40(5): 565–79. 10.1016/j.jemermed.2009.11.007 20133103

[38] Judy K, Veselik J. Workplace violence: a survey of paediatric residents. Occup Med (Lond). 2009;59(7): 472–5. 10.1093/occmed/kqp068 19460874

[39] Zainal N, Rasdi I, Saliluddin SM. The risk factors of workplace violence among healthcare workers in public hospital. Mal J Med Health Sci. 2018;14(SP2): 120–127.

[40] Coles J, Koritsas S, Boyle M, Stanley J. GPs, violence and work performance—'just part of the job?'. Aust Fam Physician. 2007;36(3): 189–91. 17339990

[41] Garrick A, Mak AS, Cathcart S, Winwood PC, Bakker AB., Lushington K. Non-work time activities predicting teachers’ work-related fatigue and engagement: an effort-recovery approach. Aust Psychol. 2018;53: 243–252. 10.1111/ap.12290

[42] Mohd Fauzi MF, Mohd Yusoff H, Mat Saruan NA, Muhamad Robat R, Abdul Manaf MR, Ghazali M. Fatigue and recovery among Malaysian doctors: the role of work-related activities during non-work time. BMJ Open. 2020;10:e036849 10.1136/bmjopen-2020-036849 32978189 PMC7520834

[43] Morgado FFR, Meireles JFF, Neves CM. Amaral ACS, Ferreira MEC. Scale development: ten main limitations and recommendations to improve future research practices. Psicol Refl Crít. 2018;30: 3 10.1186/s41155-016-0057-1 32025957 PMC6966966

[44] Boateng GO, Neilands TB, Frongillo EA, Melgar-Quiñonez HR, Young SL. Best practices for developing and validating scales for health, social, and behavioral research: a primer. Front Public Health. 2018;6:149 10.3389/fpubh.2018.00149 29942800 PMC6004510

[45] DeVellis RF. Scale development: theory and applications. 2nd ed Newbury Park, United States: Sage Publications; 2003.

[46] Worthington RL, Whittaker TA. Scale development research: a content analysis and recommendations for best practices. Couns Psychol. 2006;34(6): 806–838. 10.1177/0011000006288127

[47] Armstrong TS, Cohen MZ, Eriksen L, Cleeland C. Content validity of self-report measurement instruments: an illustration from the development of the Brain Tumor Module of the M.D. Anderson Symptom Inventory. Oncol Nurs Forum. 2005;32(3):669–76. 10.1188/05.ONF.669-676 15897941

[48] Haynes SN, Richard DCS, Kubany ES. Content validity in psychological assessment: A functional approach to concepts and methods. Psychological Assessment. 1995;7(3): 238–247. 10.1037/1040-3590.7.3.238

[49] Davis LL. Instrument review: Getting the most from a panel of experts. Applied Nursing Research. 1992;5(4):194–7. 10.1016/S0897-1897(05)80008-4

[50] Zamanzadeh V, Ghahramanian A, Rassouli M, Abbaszadeh A, Alavi-Majd H, Nikanfar AR. Design and implementation content validity study: development of an instrument for measuring patient-centered communication. J Caring Sci. 2015;4(2):165–178. 10.15171/jcs.2015.017 26161370 PMC4484991

[51] Lynn MR. Determination and quantification of content validity. Nursing Research. 1986; 35: 382–385. 3640358

[52] Polit DF, Beck CT, Owen SV. Is the CVI an acceptable indicator of content validity? Appraisal and recommendations. Res Nurs Health. 2007;30(4):459–67. 10.1002/nur.20199 17654487

[53] Shi J, Mo X, Sun Z. [Content validity index in scale development]. Zhong Nan Da Xue Xue Bao Yi Xue Ban. 2012 2;37(2):152–5. Chinese. 10.3969/j.issn.1672-7347.2012.02.007 22561427

[54] Yamada J, Stevens B, Sidani S, Watt-Watson J, de Silva N. Content validity of a process evaluation checklist to measure intervention implementation fidelity of the EPIC intervention. Worldviews Evid Based Nurs. 2010;7(3):158–64. 10.1111/j.1741-6787.2010.00182.x 20180940

[55] Rodrigues IB, Adachi JD, Beattie KA, MacDermid JC. Development and validation of a new tool to measure the facilitators, barriers and preferences to exercise in people with osteoporosis. BMC Musculoskelet Disord. 2017; 18(1):540 10.1186/s12891-017-1914-5 29258503 PMC5738121

[56] Collins D. Pretesting survey instruments: An overview of cognitive methods. Qual Life Res 12, 229–238 (2003). 10.1023/A:1023254226592 12769135

[57] Hair JF, Black WC, Babin BJ, Anderson RE. Exploratory factor analysis In: Hair JF, Black WC, Babin BJ, Anderson RE, editors. Multivariate Data Analysis. 7th ed England, UK: Pearson; 2014 pp. 89–149.

[58] Samuels P. Advice on exploratory factor analysis: technical report. 2017. https://www.researchgate.net/publication/319165677_Advice_on_Exploratory_Factor_Analysis

[59] Costello AB, Osborne JW. Best practices in exploratory factor analysis: four recommendations for getting the most from your analysis. Pract Assess Res Eval. 2005;10(7): 1–9. 10.7275/jyj1-4868

[60] Field A. Discovering statistics using SPSS. 4th ed London, United Kingdom: SAGE; 2013.

[61] Kaiser HF. An index of factorial simplicity. Psychometrika. 1974; 39(1): 31–36. 10.1007/BF02291575

[62] Streiner DL. Figuring out factors: the use and misuse of factor analysis. Can J Psychiatry. 1994;39(3): 135–40. 10.1177/070674379403900303 8033017

[63] Wendsche J, Lohmann-Haislah A. A Meta-Analysis on Antecedents and Outcomes of Detachment from Work. Front Psychol. 2017;7:2072 10.3389/fpsyg.2016.02072 28133454 PMC5233687

[64] Bakker AB, Demerouti E, Oerlemans W, Sonnentag S. Workaholism and daily recovery: A day reconstruction study of leisure activities. Journal of Organizational Behavior. 2013;34(1), 87–107. 10.1002/job.1796

[65] Mojza EJ, Sonnentag S, Bornemann C. Volunteer work as a valuable leisure-time activity: A day-level study on volunteer work, non-work experiences, and well-being at work. J Occup Organ Psychol. 2011;84(1): 123–152. 10.1348/096317910X485737

[66] Cropley M, Zijlstra FRH. Work and rumination In: Langan-Fox J, Cooper CL, editors. Handbook of Stress in the Occupations. Cheltenham, PA, United States: Edward Elgar Publishing Ltd; 2011 pp. 487–503

[67] Smith JM, Alloy LB. A roadmap to rumination: a review of the definition, assessment, and conceptualization of this multifaceted construct. Clin Psychol Rev. 2009;29(2): 116–28. 10.1016/j.cpr.2008.10.003 19128864 PMC2832862

[68] Alloy LB, Abramson LY, Hogan ME, Whitehouse WG, Rose DT, Robinson MS, et al The Temple-Wisconsin Cognitive Vulnerability to Depression Project: lifetime history of axis I psychopathology in individuals at high and low cognitive risk for depression. J Abnorm Psychol. 2000;109(3): 403–18. 11016110

[69] Mellings TM, Alden LE. Cognitive processes in social anxiety: the effects of self-focus, rumination and anticipatory processing. Behav Res Ther. 2000;38(3):243–57. 10.1016/s0005-7967(99)00040-6 10665158

[70] Edwards SL, Rapee RM, Franklin J. Postevent rumination and recall bias for a social performance event in high and low socially anxious individuals. Cognit Ther Res. 2003; 27: 603–617. 10.1023/A:1026395526858

[71] Zedeck S, Mosier KL. Work in the family and employing organization. Am Psychol. 1990;45(2): 240–51. 10.1037//0003-066x.45.2.240 2178504

[72] Mennino SF, Rubin BA, Brayfield A. Home-to-job and job-to-home spillover: the impact of company policies and workplace culture. Sociol Q. 2005;46(1): 107–135. 10.1111/j.1533-8525.2005.00006.x

[73] Grzywacz JG, Marks NF. Reconceptualizing the work-family interface: an ecological perspective on the correlates of positive and negative spillover between work and family. J Occup Health Psychol. 2000;5(1):111–26. 10.1037//1076-8998.5.1.111 10658890

[74] Ashforth BE, Kreiner GE, Fugate M. All in a day’s work: boundaries and micro role transitions. Acad Manag Rev. 2000; 25: 472–491. 10.2307/259305

[75] Clark SC. Work/family Border theory: a new theory of work/family balance. Hum Relat. 2000; 53: 747–770. 10.1177/0018726700536001

[76] Kreiner GE. Consequences of work–home segmentation or integration: a person-environment fit perspective. J Organ Behav. 2006; 27: 485–507. 10.1002/job.386

[77] Hobfoll SE. Conservation of resources. A new attempt at conceptualizing stress. Am Psychol. 1989;44(3):513–24. 10.1037//0003-066x.44.3.513 2648906

[78] Jablin FM. Superior-subordinate communication: the state of the art. Psychol Bull. 1979;86: 1201–1222. 10.1037/0033-2909.86.6.1201

[79] Winska J. Influence of superior-subordinate communication on employee satisfaction. J Posit Manag. 2010;1(1): 110–124. 10.12775/JPM.2010.009

[80] Harriman B. Up and down the communication ladder. Harv Bus Rev. 1974; 144–145.

