# Supplementary material for: Development and validation of Work-Related Activities during Non-Work Time Scale (WANTS) for doctors
Source: PLoS One. 2020 Nov 18;15(11):e0241577. doi: 10.1371/journal.pone.0241577 (PMC7673515; doi:10.1371/journal.pone.0241577)
Supplement: S1 Questionnaire — Bulatkan pada satu nombor antara 0 hingga 6 yang paling menggambarkan kekerapan anda melakukan aktiviti-aktiviti berkaitan kerja tersebut ketika bukan waktu bekerja. (The following are statements regarding work-related activities that you do beyond official work hours. Please circle on the number between 0 (never) to 6 (daily) that represents the frequency you do work-related activities beyond official work hours). (DOCX) [file pone.0241577.s001.docx]

**S1 Questionnaire. Berikut ialah kenyataan tentang aktiviti-aktiviti berkaitan kerja yang anda lakukan ketika bukan waktu bekerja. Bulatkan pada satu nombor antara 0 hingga 6 yang paling menggambarkan kekerapan anda melakukan aktiviti-aktiviti berkaitan kerja tersebut ketika bukan waktu bekerja.** *(The following are statements regarding work-related activities that you do beyond official work hours. Please circle on the number between 0 (never) to 6 (daily) that represents the frequency you do work-related activities beyond official work hours.)*

| **AKTIVITI-AKTIVITI BERKAITAN KERJA KETIKA BUKAN WAKTU BEKERJA**  *(WORK-RELATED ACTIVITIES DURING NON-WORK TIME)* | **KEKERAPAN** *(FREQUENCY)* | | | | | | |
| --- | --- | --- | --- | --- | --- | --- | --- |
|  | **Tidak pernah**  *(Never)* | **Kurang sekali dalam sebulan**  *(Less than once a month)* | **Sekali dalam sebulan**  *(Once a month)* | **Lebih sekali dalam sebulan**  *(More than once a month)* | **Sekali dalam seminggu**  *(Once a week)* | **Lebih sekali dalam seminggu**  *(More than once a week)* | **Setiap hari**  *(Daily)* |
| **Menghadiri latihan berkaitan kerja di tempat kerja**  *(Attending work-related upskill training at workplace)* | 0 | 1 | 2 | 3 | 4 | 5 | 6 |
| **Menghadiri mesyuarat rasmi berkaitan kerja secara fizikal di tempat kerja**  *(Attending formal, work-related meeting physically at the workplace)* | 0 | 1 | 2 | 3 | 4 | 5 | 6 |
| **Menghadiri mesyuarat rasmi berkaitan kerja secara maya**  *(Attending formal, work-related meeting virtually)* | 0 | 1 | 2 | 3 | 4 | 5 | 6 |
| **Perbincangan tidak rasmi berkaitan kerja bersama rakan sekerja secara bersemuka**  *(Informal, work-related discussions with colleagues at the workplace)* | 0 | 1 | 2 | 3 | 4 | 5 | 6 |
| **Perbincangan tidak rasmi berkaitan kerja bersama rakan sekerja secara maya**  *(Informal, work-related discussions with colleagues virtually)* | 0 | 1 | 2 | 3 | 4 | 5 | 6 |
| **Berborak hal-hal kerja bersama pasangan / suami / isteri secara bersemuka**  *(Informal face to face conversations about work with spouse or partner)* | 0 | 1 | 2 | 3 | 4 | 5 | 6 |
| **Berborak hal-hal kerja bersama pasangan / suami / isteri secara maya**  *(Informal conversation about work with spouse or partner virtually)* | 0 | 1 | 2 | 3 | 4 | 5 | 6 |
| **Berborak hal-hal kerja bersama ibu/bapa secara bersemuka**  *(Face to face conversations about work with parents)* | 0 | 1 | 2 | 3 | 4 | 5 | 6 |
| **Berborak hal-hal kerja bersama ibu / bapa secara maya**  *(Virtual conversations about work with parents)* | 0 | 1 | 2 | 3 | 4 | 5 | 6 |
| **Komunikasi bersama pesakit / klien secara bersemuka**  *(Face to face communications with patients / clients)* | 0 | 1 | 2 | 3 | 4 | 5 | 6 |
| **Komunikasi bersama pesakit / klien secara maya**  *(Virtual communications with patients / clients)* | 0 | 1 | 2 | 3 | 4 | 5 | 6 |
| **Melakukan tugasan tempat kerja di tempat kerja selepas waktu bekerja rasmi**  *(Performing official tasks at the workplace beyond official work hours)* | 0 | 1 | 2 | 3 | 4 | 5 | 6 |
| **Melakukan tugasan tempat kerja di rumah**  *(Doing official tasks at home)* | 0 | 1 | 2 | 3 | 4 | 5 | 6 |
| **Menerima tugasan daripada majikan melalui panggilan telefon**  *(Formal work assignments by employer via telephone call)* | 0 | 1 | 2 | 3 | 4 | 5 | 6 |
| **Menerima tugasan daripada majikan melalui pesanan teks / emel**  *(Formal work assignments by employer via text or email)* | 0 | 1 | 2 | 3 | 4 | 5 | 6 |
| **Mengendalikan emel / laman sesawang / laman sosial berkaitan kerja**  *(Handling work-related email / website / social media)* | 0 | 1 | 2 | 3 | 4 | 5 | 6 |
| **Memikirkan kerja-kerja di tempat kerja yang masih tertangguh**  *(Work-related thoughts on unfinished formal task)* | 0 | 1 | 2 | 3 | 4 | 5 | 6 |
| **Memikirkan kerja-kerja di tempat kerja yang bakal diterima**  *(Work-related thoughts on upcoming formal task and assignments)* | 0 | 1 | 2 | 3 | 4 | 5 | 6 |
| **Memikirkan hal-hal pesakit / klien di tempat kerja**  *(Work-related thoughts on patients / clients)* | 0 | 1 | 2 | 3 | 4 | 5 | 6 |
| **Memikirkan peristiwa dimarahi atau dikasari yang berlaku di tempat kerja**  *(Work-related thoughts on incident of being scolded or violated at work)* | 0 | 1 | 2 | 3 | 4 | 5 | 6 |
| **Memikirkan kesilapan yang dibuat sama ada secara sengaja atau tidak sengaja di tempat kerja**  *(Work-related thoughts on mistakes being done at work either deliberately or unintentionally)* | 0 | 1 | 2 | 3 | 4 | 5 | 6 |
| **Lokum di fasiliti kesihatan kerajaan**  *(Locum at government health facilities)* | 0 | 1 | 2 | 3 | 4 | 5 | 6 |
| **Lokum di fasiliti kesihatan swasta**  *(Locum at private health facilities)* | 0 | 1 | 2 | 3 | 4 | 5 | 6 |
